# Change in blood pressure variability in patients with acute ischemic stroke and its effect on early neurologic outcome

**DOI:** 10.1371/journal.pone.0189216

**Published:** 2017-12-18

**Authors:** Jihoon Kang, Jeong-Ho Hong, Min Uk Jang, Nack Cheon Choi, Ji Sung Lee, Beom Joon Kim, Moon-Ku Han, Hee-Joon Bae

**Affiliations:** 1 Department of Neurology, Seoul National University Bundang Hospital, Seoul National University, Seongnam, Republic of Korea; 2 Department of Neurology, Dongsan Medical Center, Keimyung University School of Medicine, Daegu, Republic of Korea; 3 Department of Neurology, Dongtan Sacred Heart Hospital, Hallym University, School of Medicine, Dongtan, Republic of Korea; 4 Department of Neurology, Gyneongsang Institute for Neuroscience, Gyengsang National University College of Medicine, Jinju, Republic of Korea; 5 Clinical Research Center, Asan Medical Center, Ulsan University School of Medicine, Seoul, Republic of Korea; The University of Tokyo, JAPAN

## Abstract

**Background:**

How short-term blood pressure variability (BPV) is affected in the acute stage of ischemic stroke and whether BPV is associated with early neurologic outcomes remains unclear.

**Methods:**

Patients who admitted for ischemic stroke within 24 h of symptom onset were consecutively identified between January 2010 and January 2015. BP profiles measured in real-time were summarized into short-term, 24-h time intervals, based on standard deviation (SD) and mean of systolic BP (SBP_SD_) during the first 3 days. The primary outcome was daily assessment of early neurological deterioration (END). The associations between short-term SBP_SD_ values and the secular trend for primary outcome were examined.

**Results:**

A total of 2,545 subjects (mean age, 67.1 ± 13.5 years old and median baseline National Institutes of Health Stroke Scale score, 3) arrived at the hospital an average of 6.1 ± 6.6 h after symptom onset. SBP_SD_ values at day 1 (SD#D1), SD#D2, and SD#D3 were 14.4 ± 5.0, 12.5 ± 4.5, and 12.2 ± 4.6 mmHg, respectively. Multivariable analyses showed that SD#D2 was independently associated with onset of END at day 2 (adjusted odds ratio, 1.08; 95% confidence interval, 1.03–1.13), and SD#D3 was independently associated with END#D3 (1.07, 1.01–1.14), with adjustments for predetermined covariates, SBP_mean_, and interactions with daily SBP_SD_.

**Conclusion:**

Short-term BPV changed and stabilized from the first day of ischemic stroke. Daily high BPV may be associated with neurological deterioration independent of BPV on the previous day.

## Introduction

Blood pressure (BP) generally goes through abrupt and rapid changes in the acute stage of ischemic stroke; approximately 80% of stroke patients initially have an elevated BP, which subsequently stabilizes within a few days.[1,2] This abrupt reactive response has been linked to stroke prognosis, that is, the mean value of BP has been established as an important prognostic factor for cardiovascular outcome.

However, BP variability (BPV), another element comprising the underlying theoretical true BP, has yielded inconsistent results.[3] Some studies suggested that high BPV in the acute stage of ischemic stroke exerted an adverse influence on the progress of early neurologic status.[4–7] High BPV during the first 72 h after stroke onset is associated with occurrence of hemorrhagic transformation and growth of ischemic lesion size, which subsequently increase the risk of poor outcome. However, a recent observational study reported the contradictory finding that an increase in BPV in acute stage had no specific association with 3-month outcome in a large stroke population.[8] Post-hoc analysis from the Controlling Hypertension and Hypotension Immediately Post Stroke (CHHIPS) study and Continue or Stop Post-Stroke Antihypertensives Collaborative Study (COSSACS) supported the this neutral effect of BPV in the acute stage.[9]

Several conditions could account for these conflicting results. First, the time interval for measurement of BPV must be standardized appropriately. Since BP changes rapidly in the acute stage of ischemic stroke, BPV might be variably estimated according to the measurement time interval.[10] In this situation, a short-term period defined as 24 h would appropriately reflect physiological and neurohumoral conditions rather than use of another measurement interval, such as an ultra-short-term (beat-to-beat), or long-term (day-by-day) interval.[11] Second, short-term BPV observation for 3 consecutive days might be advantageous for determination of the time course of BP during the acute reactive response and subsequent stabilization.[12] Third, the acute stage of ischemic stroke is a high-risk period for neurologic worsening, and is a major factor affecting stroke outcome in addition to baseline neurologic severity.[13–15] Therefore, it would be clinically meaningful to investigate daily short-term BPV in the acute stage and to examine its effect on early neurologic deterioration (END).

In this study, daily short-term BPV during the first 3 days of acute ischemic stroke was examined in hospitalized patients who arrived within 24 h of symptom onset. END was prospectively monitored and documented during the same period.[14,16] We aimed to investigate whether short-term BPV and the changing BPV trend affected the onset of END in the acute stage of ischemic stroke.

## Methods

### Standard protocol approval and patient consent

This study was approved by the local Institutional Review Board of Seoul National University Bundang Hospital with a waiver of informed consent because of its retrospective and observational design, which posed no potential harm to enrolled patients. Prior to analysis, all medical records and personal information were anonymized and de-identified.

### Subjects and data collection

Patients admitted for ischemic stroke at Seoul National University Bundang Hospital, Republic of Korea, who arrived within 24 h of symptom onset were consecutively identified between January 2010 and January 2015. Ischemic stroke is defined as a sudden onset neurologic dysfunction caused by focal cerebral, spinal or retinal infarction confirmed by imaging studies.[17] Demographic and stroke information were collected by reviewing the institution’s embedded electronic health records (EHRs) and the hospital’s prospective stroke registry.[18]

BP data of enrolled subjects in the acute stage, defined as the time from stroke onset to 72 h after onset, were downloaded from the EHR. Routine hospital management strategy required patients to be admitted to the stroke unit or intensive care unit, to monitor BP according to the physician’s decision, based on current stroke guidelines and the patient’s condition.[19]

END during hospitalization was prospectively documented, with a final determination made through a weekly consensus meeting, as part of a quality-of-care monitoring and improvement program for hospitalized stroke patients. Neurologic deterioration was operationally defined as worsening by 2 points or more in the total National Institutes of Health Stroke Scale (NIHSS) score. Neurologic deterioration caused by increased intracranial pressure or comorbid medical illness was excluded [16]. END at day 1, day 2, and day 3 (END#D1, END#D2, and END#D3) were separately estimated as a primary outcome. A 3-month poor outcome was defined as a modified Rankin Scale (mRS) score between 3 and 6, and was captured as a secondary outcome. Covariates were predetermined by consideration of previous research and investigator consensus, and included age, diabetes mellitus, baseline NIHSS score, time to arrival, stroke subtype, history of stroke, and recanalization therapy.[14,15,20–26]

### Statistical analysis

Collected BP parameters were summarized into mean SBP (SBP_mean_) at day 1, day 2, and day 3 (M#D1, M#D2, and M#D3) and SD of SBP (SBP_SD_) at day 1, day 2, and day 3 (SD#D1, SD#D2, and SD#D3). Their associations with predetermined baseline characteristics were investigated as appropriate.

**Fig 1:**
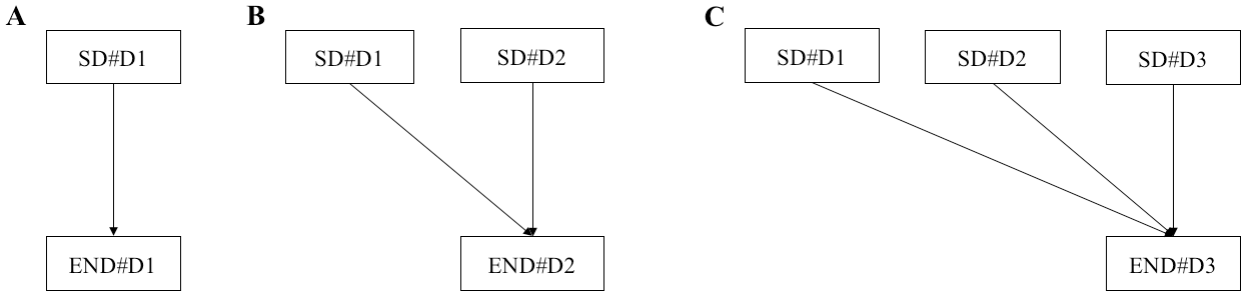
Statistical association between SBP_SD_ and primary outcomes. Daily SBP_SD_ values were investigated for END#D1(A), END#D2 (B), and END#D3 (C). In multivariable analysis, the interaction term SD#D1 × SD#D2 was included in model B and SD#D1 × SD#D2, SD#D1 × SD#D3, SD#D2 × SD#D3, and SD#D1 × SD#D2 × SD#D3 were included in model C.

The scheme for analyzing statistical association between SBP_SD_ and primary outcome is shown (Fig 1). In bivariate analysis, the associations between daily SBP_SD_ and primary outcomes were compared. The same analyses were performed for SBP_mean_. Multivariable logistic regression models were structured using daily short-term BP parameters, with adjustments for pre-selected confounders. These models included interaction effects between the present and previous day for END. We estimated the adjusted odds ratios of daily SD as the secondary outcome.

## Results

### Baseline characteristics

A total of 2,454 patients were enrolled. Their mean age was 67.1 ± 13.5 years old and median baseline NIHSS score was 3 (interquartile range, IQR, 1–9). They arrived at the hospital an average of 6.1 ± 6.6 h after stroke onset, and the median numbers of BP measurements in the acute stage were 26 (IQR, 22–30), 24 (22–26), and 22 (9–25) at day 1, 2, and 3, respectively.

The SD#D1, SD#2, and SD#3 values were 14.4 ± 5.0, 12.5 ± 4.5, and 12.2 ± 4.6 mmHg, and the mean#D1 (M#D1), M#2, and M#3 values were 134.5 ± 16.7, 132.9 ± 16.7, and 134.7 ± 16.7 mmHg, respectively. The comparisons of baseline characteristics according to SD#1, SD#2, and SD#3 demonstrated consistent associations with age, sex, baseline NIHSS score, hypertension, and stroke subtype (Table 1).

**Table 1:** Association of baseline characteristics with daily SBP_SD_.

Variables	SD#D1	P	SD#D2	P	SD#D3	P
Age, y, mean ± SD	ρ = 0.14	< 0.001	ρ = 0.26	< 0.001	ρ = 0.20	< 0.001
Sex		< 0.001		< 0.001		0.01
Male (n = 1458)	14.0 ± 5.3		12.2 ± 4.4		12.0 ± 4.6	
Female (n = 983)	15.1 ± 5.3		13.0 ± 4.7		12.5 ± 5.0	
Baseline NIHSS score, median (IQR)	σ = 0.05	0.01	σ = 0.10	< 0.001	σ = 0.09	< 0.001
Recanalization therapy		0.79		0.35		0.02
None (n = 1883)	14.4 ± 5.0		12.5 ± 4.4		12.3 ± 4.8	
IVT–only (n = 192)	14.8 ± 4.8		12.7 ± 4.4		12.4 ± 4.2	
EVT–only (n = 179)	14.4 ± 5.9		12.1 ± 5.0		11.4 ± 4.3	
Combined treatment (n = 187)	14.6 ± 5.0		12.1 ± 4.9		11.7 ± 4.1	
History of stroke		0.15		< 0.001		0.09
Yes (n = 527)	14.8 ± 5.3		13.3 ± 4.8		12.5 ± 4.5	
No	14.5 ± 5.0		12.3 ± 4.4		12.1 ± 4.7	
Hypertension		< 0.001		< 0.001		< 0.001
Yes (n = 1705)	14.8 ± 5.0		13.0 ± 4.5		12.7 ± 4.5	
No	13.6 ± 4.9		11.2 ± 4.3		11.2 ± 4.8	
Diabetes mellitus		0.10		< 0.001		< 0.001
Yes (n = 711)	14.7 ± 5.1		13.2 ± 4.7		12.8 ± 4.8	
No	14.3 ± 5.0		12.2 ± 4.4		12.0 ± 4.5	
Dyslipidemia		0.99		0.35		0.64
Yes (n = 767)	1.45 ± 5.0		12.6 ± 4.5		12.3 ± 4.5	
No	14.5 ± 5.0		12.4 ± 4.5		12.2 ± 4.7	
Atrial fibrillation		0.56		0.03		0.53
Yes (n = 593)	14.5 ± 5.1		12.8 ± 4.8		12.3 ± 4.5	
No	14.4 ± 5.0		12.4 ± 4.4		12.2 ± 4.7	
Stroke subtypes		< 0.001		< 0.001		< 0.001
Small vessel occlusion (n = 399)	15.4 ± 5.4		12.7 ± 4.4		12.8 ± 4.8	
Large artery atherosclerosis (n = 787)	14.5 ± 5.0		13.0 ± 4.4		12.7 ± 4.7	
Cardioembolism (n = 720)	14.3 ± 5.0		12.5 ± 4.6		12.2 ± 4.5	
Other determined (n = 107)	12.3 ± 5.0		10.0 ± 3.3		10.1 ± 3.8	
Undetermined (n = 441)	14.2 ± 4.7		12.5 ± 4.5		11.3 ± 4.4	

Values are mean ± SD (mmHg) except for interval variables describing correlation coefficient values, with ρ for parametric and σ for non-parametric variables. P values were obtained by t-test, ANOVA test, Pearson correlation test, and Spearman’s correlation test as appropriate. NIHSS = National Institutes of Health Stroke Scale, SD = standard deviation, IQR = interquartile range, IVT = intravenous thrombolysis, and EVT = endovascular treatment.

During hospitalization, 256 (10.4%) subjects experienced END. Of these, 94.5% occurred in the first 3 days; END#D1, END#D2, and END#D3 occurred in 4.1%, 3.5%, and 1.5%, respectively.

### BP parameters and primary outcome

**Fig 2:**
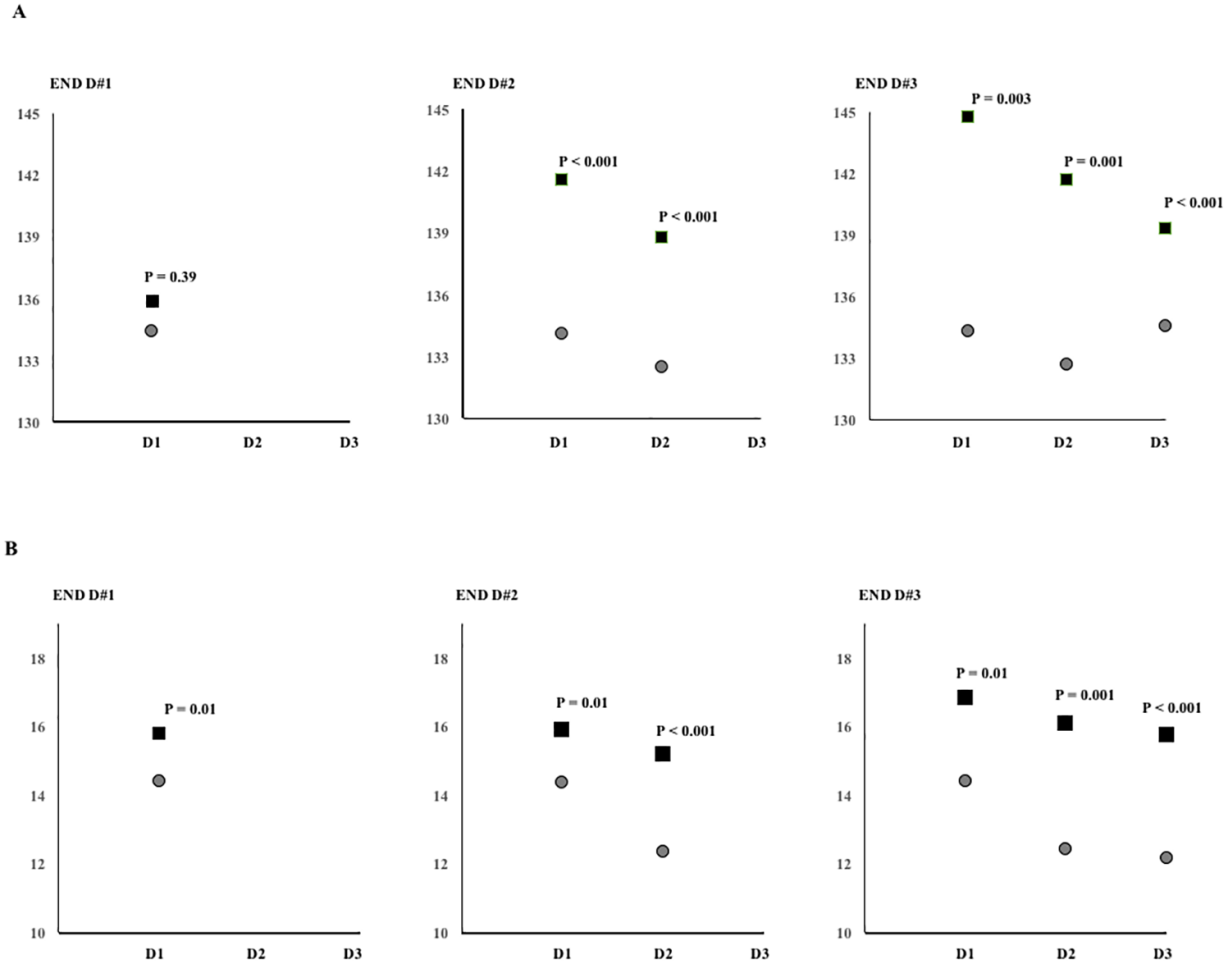
Comparisons of daily SBP parameters according to daily END. The daily means (A) and standard deviations (B) of SBP are shown as patients with END (box) and without END (circle).

In bivariate analysis, associations between SBP parameters and primary outcome were demonstrated (Fig 2).

The M#D1 was not significantly associated with END#D1. The M#D1 and M#D2 showed significant associations with END#D2; M#D1 and M#D2, but not M#D3, showed significant associations with END#D3 (Fig 2A). SD#D1 values were significantly associated with END#D1; SD#D1 and SD#D2 showed significant associations with END#D2; SD#D1, SD#D2, and SD#D3 showed significant associations with END#D3 (Fig 2B, Ps<0.05)

The multivariable logistic regression models showed that SD#D2 increased the odds for END#D2 (adjusted odds ratio, 1.08; 95% confidence interval, 1.03–1.13), independent of the predetermined covariates, previous day BP parameters, and interaction between SD#D1 and SD#D2. SD#D3 independently increased the odds for END#D3 (1.07, 1.01–1.14, Table 2).

**Table 2:** Associations between daily SBP_SD_ and END.

SBP parameters	Crude analysis			Multivariable analysis		
	END#D1	END#D2	END#D3	END#D1	END#D2	END#D3
SD#D1	1.05 (1.01–1.09)	1.04 (1.01–1.09)	1.08 (1.02–1.13)	1.03 (1.00–1.07)	0.98 (0.94–1.03)	1.01 (0.94–1.07)
SD#D2		1.09 (1.06–1.13)	1.12 (1.07–1.18)		1.08 (1.03–1.13)	1.05 (0.98–1.12)
SD#D3			1.11 (1.06–1.16)			1.07 (1.01–1.14)
					P of SD#1 × SD#2 = 0.13	P of SD#1 × SD#2 = 0.95
						P of SD#1 × SD#3 = 0.06
						P of SD#2 × SD#3 = 0.77
						P of SD#1 × SD#2 × SDSD#3 = 0.76
Mean#D1	1.01 (0.99–1.02)	1.02 (1.01–1.03)	1.03 (1.02–1.05)	1.01 (1.00–1.03)	1.02 (1.00–1.04)	1.03 (1.00–1.06)
Mean#D2		1.02 (1.00–1.03)	1.03 (1.01–1.05)		1.00 (0.98–1.02)	1.01 (0.97–1.04)
Mean#D3			1.02 (1.00–1.04)			0.99 (0.96–1.02)

Values were crude odds ratios (95% confidence interval) and were estimated using the logistic regression model. Multivariable analyses included BP parameters with adjustments for age, time to arrival, recanalization therapy, hypertension, diabetes, atrial fibrillation, baseline NIHSS score and stroke subtypes.

### BP parameters and functional outcome

The associations between SBP parameters and 3-month functional outcome were summarized (Table 3). SD#D2 and SD#D3 were independently associated with 3-month poor outcome, with every SD increase of 1 mmHg at day 2 and day 3 increasing the odds for poor outcome by 7% and 4%, respectively.

**Table 3:** Daily SDs of SBP and 3-month functional outcome.

SBP parameters	OR (95% CI) for poor outcome
SD#D1	1.00 (0.97–1.03)
SD#D2	1.07 (1.04–1.11)
SD#D3	1.04 (1.01–1.07)
Mean#D1	1.01 (1.00–1.02)
Mean#D2	1.01 (0.99–1.02)
Mean#D3	0.99 (0.97–1.03)

Logistic regression models were used to calculate the odds ratios (95% confidence interval), with adjustments for age, time to arrival, recanalization therapy, hypertension, diabetes, atrial fibrillation, baseline NIHSS score, and stroke subtype.

## Discussion

This study showed that BPV in the acute stage of ischemic stroke generally decreased and stabilized. Under these circumstances in ischemic stroke, daily estimation of BPV would be helpful in determining the risk of END.

The 24-h time window, short-term BPV in patients with ischemic stroke decreased from 14.4 mmHg to 12.2 mmHg during the acute stage. As the mean value of SBP showed a similar range during this period, stabilization of BP was consistent with the decrease in BPV, in other words, fluctuation of BP decreased over time.

A high short-term BPV, meanwhile, showed a significant association with onset of neurologic deterioration, independent of the mean BP and BPV on the previous day. Interestingly, daily BPV was not affected by BPV on the previous day. This result shows the need for serial BP monitoring and estimation of BPV. High BPV would assert a harmful effect to maintain the hemodynamic stability in patients impaired the cerebral autoregulation, which, in turn, increase the risk of lesion size growth and new lesions.[27]

BP fluctuates continuously to maintain physiological homeostasis in response to various external and internal conditions, and has been used as an important indicator to track major clinical events, such as cardiac arrest.[28,29] Our findings show how BPV obtained from real-time clinical monitoring can be used in patients with ischemic stroke.

Even though patients with ischemic stroke had similar baseline stroke severity, they presented with various medical and neurologic conditions through acute stage that led to different stroke outcomes. This showed the clinical value of an additional indicator or marker of progress for stroke. BP reflects the change in physiologic conditions and BP monitoring can rapidly detect changes in the patient’s condition. END also showed a time-dependent trend, with most changes occurring in the first few days. Since conventional risk factors for END, such as age, baseline stroke severity, cerebral arterial steno-occlusive lesion,[22] and diabetes mellitus[30] were mostly determined at initial work up process, they cannot be used to assess the change in a patient’s condition.

Previous studies used longer intervals and reported a 14.8–16.5 mmHg SBP_SD_ in the acute stage of ischemic stroke.[5,8,27] Since short-term BPV decreased over time, 3-day estimation would yield generally higher values than daily estimation, and the latter method would more promptly show the changing course in stroke victims. Generally, 24-h estimation of BPV is advantageous for demonstrating vascular dysfunction,[31,32] cardiac dysfunction,[33] reduced arterial reflexes, and behavioral and emotional responses.[12] These features of high BPV would demonstrate the detrimental effect on stroke prognosis.

There are several limitations. First, this study was retrospectively conducted using a single hospital database, and had a risk of bias. However, our subjects were consecutively identified from a prospective stroke registry and large amounts of BP data would increase the statistical power. Second, this study only showed an association between BPV and END on a daily basis, and could not explain a causal relationship. Because we aimed to identify the course and impact of short-term BPV, a different study design and more precise analytic tools are needed. Third, BP data were obtained from real-time clinical information and the measurement time interval and numbers were not predetermined. Because median numbers of BP measurements ranged from 22 to 26 times per day and stroke management practice generally measures BP every hour in the acute stage, our estimated values might be similar to those obtained with 24-h ambulatory BP monitoring.

## References

[1] Willmot M, Leonardi-Bee J, Bath PMW. High Blood Pressure in Acute Stroke and Subsequent Outcome: A Systematic Review. Hypertension. 2004;43: 18–24. doi: 10.1161/01.HYP.0000105052.65787.35 14662649 10.1161/01.HYP.0000105052.65787.35

[2] Goldstein LB. Blood Pressure Management in Patients With Acute Ischemic Stroke. Hypertension. 2004;43: 137–141. doi: 10.1161/01.HYP.0000113297.76013.51 14718352 10.1161/01.HYP.0000113297.76013.51

[3] Rothwell PM. Limitations of the usual blood-pressure hypothesis and importance of variability, instability, and episodic hypertension. The Lancet. Elsevier Ltd; 2010;375: 938–948. doi: 10.1016/S0140-6736(10)60309-1 10.1016/S0140-6736(10)60309-1 20226991

[4] Delgado-Mederos R, Ribo M, Rovira A, Rubiera M, Munuera J, Santamarina E, et al Prognostic significance of blood pressure variability after thrombolysis in acute stroke. Neurology. 2008;71: 552–558. doi: 10.1212/01.wnl.0000318294.36223.69 18550860 10.1212/01.wnl.0000318294.36223.69

[5] Ko Y, Park JH, Yang MH, Ko S-B, Han MK, Oh CW, et al The significance of blood pressure variability for the development of hemorrhagic transformation in acute ischemic stroke. Stroke. 2010;41: 2512–2518. doi: 10.1161/STROKEAHA.110.595561 20947842 10.1161/STROKEAHA.110.595561

[6] Endo K, Kario K, Koga M, Nakagawara J, Shiokawa Y, Yamagami H, et al Impact of early blood pressure variability on stroke outcomes after thrombolysis: the SAMURAI rt-PA Registry. Stroke. 2013;44: 816–818. doi: 10.1161/STROKEAHA.112.681007 23329210 10.1161/STROKEAHA.112.681007

[7] Dawson SL, Manktelow BN, Robinson TG, Panerai RB, Potter JF. Which Parameters of Beat-to-Beat Blood Pressure and Variability Best Predict Early Outcome After Acute Ischemic Stroke? Stroke. Lippincott Williams & Wilkins; 2000;31: 463–468. doi: 10.1161/01.STR.31.2.463 10.1161/01.str.31.2.463 10657423

[8] Fukuda K, Kai H, Kamouchi M, Hata J, Ago T, Nakane H, et al Day-by-Day Blood Pressure Variability and Functional Outcome After Acute Ischemic Stroke: Fukuoka Stroke Registry. Stroke. 2015;46: 1832–1839. doi: 10.1161/STROKEAHA.115.009076 26069262 10.1161/STROKEAHA.115.009076

[9] Manning LS, Mistri AK, Potter J, Rothwell PM, Robinson TG. Short-Term Blood Pressure Variability in Acute Stroke Post Hoc Analysis of the Controlling Hypertension and Hypotension Immediately Post Stroke and Continue or Stop Post-Stroke Antihypertensives Collaborative Study Trials. Stroke. Lippincott Williams & Wilkins; 2015;46: 1518–1524. doi: 10.1161/STROKEAHA.115.009078 25908462 10.1161/STROKEAHA.115.009078

[10] Christensen H, Meden P, Overgaard K. The course of blood pressure in acute stroke is related to the severity of the neurological deficits. Acta Neurol Scand. 2002;106: 142–147. doi: 10.1034/j.1600-0404.2002.01356.x 12174173 10.1034/j.1600-0404.2002.01356.x

[11] Parati G. Blood pressure variability: its measurement and significance in hypertension. J Hypertens Suppl. 2005;23: S19–25. 15821447 10.1097/01.hjh.0000165624.79933.d3

[12] Parati G, Ochoa JE, Lombardi C, Bilo G. Assessment and management of blood-pressure variability. Nat Rev Cardiol. 2013;10: 143–155. doi: 10.1038/nrcardio.2013.1 23399972 10.1038/nrcardio.2013.1

[13] Siegler JE, Martin-Schild S. Early Neurological Deterioration (END) after stroke: the END depends on the definition. International Journal of Stroke. 2011;6: 211–212. doi: 10.1111/j.1747-4949.2011.00596.x 21557807 10.1111/j.1747-4949.2011.00596.x

[14] Seners P, Turc G, Oppenheim C, Baron J-C. Incidence, causes and predictors of neurological deterioration occurring within 24 h following acute ischaemic stroke: a systematic review with pathophysiological implications. Journal of Neurology, Neurosurgery & Psychiatry. 2015;86: 87–94. doi: 10.1136/jnnp-2014-308327 24970907 10.1136/jnnp-2014-308327

[15] Hong KS, Kang DW, Koo JS, Yu KH, Han MK, Cho YJ, et al Impact of neurological and medical complications on 3-month outcomes in acute ischaemic stroke. European Journal of Neurology. 2008;15: 1324–1331. doi: 10.1111/j.1468-1331.2008.02310.x 19049549 10.1111/j.1468-1331.2008.02310.x

[16] Kang J, Kim N, Oh CW, Kwon O-K, Jung CK, Kim WJ, et al Symptomatic steno-occlusion of cerebral arteries and subsequent ischemic events in patients with acute ischemic stroke. J Stroke Cerebrovasc Dis. 2014;23: e347–53. doi: 10.1016/j.jstrokecerebrovasdis.2013.12.028 24582792 10.1016/j.jstrokecerebrovasdis.2013.12.028

[17] Sacco RL, Kasner SE, Broderick JP, Caplan LR, Connors JJB, Culebras A, et al An updated definition of stroke for the 21st century: a statement for healthcare professionals from the American Heart Association/American Stroke Association. Stroke. 2013;44: 2064–2089. doi: 10.1161/STR.0b013e318296aeca 23652265 10.1161/STR.0b013e318296aeca PMC11078537

[18] Yoo S, Kim S, Lee S, Lee K-H, Baek R-M, Hwang H. A study of user requests regarding the fully electronic health record system at Seoul National University Bundang Hospital: challenges for future electronic health record systems. Int J Med Inform. 2013;82: 387–397. doi: 10.1016/j.ijmedinf.2012.08.004 22959193 10.1016/j.ijmedinf.2012.08.004

[19] Jauch EC, Saver JL, Adams HP, Bruno A, Connors JJB, Demaerschalk BM, et al Guidelines for the early management of patients with acute ischemic stroke: a guideline for healthcare professionals from the American Heart Association/American Stroke Association. Stroke. 2013 pp. 870–947. doi: 10.1161/STR.0b013e318284056a 23370205 10.1161/STR.0b013e318284056a

[20] Brott TG, E C Haley J, Levy DE, Barsan W, Broderick J, Sheppard GL, et al Urgent therapy for stroke. Part I. Pilot study of tissue plasminogen activator administered within 90 minutes. Stroke. Lippincott Williams & Wilkins; 1992;23: 632–640. doi: 10.1161/01.STR.23.5.632 10.1161/01.str.23.5.632 1579958

[21] Davalos A, Toni D, Iweins F, Lesaffre E, Bastianello S, Castillo J. Neurological deterioration in acute ischemic stroke: potential predictors and associated factors in the European cooperative acute stroke study (ECASS) I. Stroke. 1999;30: 2631–2636. doi: 10.1038/ncpneuro0321 10582989 10.1161/01.str.30.12.2631

[22] Siegler JE, Boehme AK, Kumar AD, Gillette MA, Albright KC, Beasley TM, et al Identification of modifiable and nonmodifiable risk factors for neurologic deterioration after acute ischemic stroke. J Stroke Cerebrovasc Dis. 2013;22: e207–13. doi: 10.1016/j.jstrokecerebrovasdis.2012.11.006 23246190 10.1016/j.jstrokecerebrovasdis.2012.11.006 PMC3690312

[23] Toyoda K, Fujimoto S, Kamouchi M, Iida M, Okada Y. Acute Blood Pressure Levels and Neurological Deterioration in Different Subtypes of Ischemic Stroke. Stroke. 2009;40: 2585–2588. doi: 10.1161/STROKEAHA.108.543587 19407233 10.1161/STROKEAHA.108.543587

[24] Stergiou GS, Ntineri A, Kollias A, Ohkubo T, Imai Y, Parati G. Blood pressure variability assessed by home measurements: a systematic review. Hypertens Res. 2014;37: 565–572. doi: 10.1038/hr.2014.2 24553366 10.1038/hr.2014.2

[25] Schutte R, Thijs L, Liu Y-P, Asayama K, Jin Y, Odili A, et al Within-subject blood pressure level—not variability—predicts fatal and nonfatal outcomes in a general population. Hypertension. 2012;60: 1138–1147. doi: 10.1161/HYPERTENSIONAHA.112.202143 23071126 10.1161/HYPERTENSIONAHA.112.202143 PMC3607229

[26] Fukui M, Ushigome E, Tanaka M, Hamaguchi M, Tanaka T, Atsuta H, et al Home blood pressure variability on one occasion is a novel factor associated with arterial stiffness in patients with type 2 diabetes. Hypertens Res. 2013;36: 219–225. doi: 10.1038/hr.2012.177 23096230 10.1038/hr.2012.177

[27] Chung JW, Kim N, Kang J, Park SH, Kim WJ, Ko Y, et al Blood pressure variability and the development of early neurological deterioration following acute ischemic stroke. Journal of Hypertension. 2015;33: 2099–2106. doi: 10.1097/HJH.0000000000000675 26237556 10.1097/HJH.0000000000000675

[28] Churpek MM, Yuen TC, Park SY, Gibbons R, Edelson DP. Using electronic health record data to develop and validate a prediction model for adverse outcomes in the wards. Critical Care Medicine. 2014;42: 841–848. doi: 10.1097/CCM.0000000000000038 24247472 10.1097/CCM.0000000000000038 PMC3959228

[29] Cuthbertson BH, Boroujerdi M, McKie L, Aucott L, Prescott G. Can physiological variables and early warning scoring systems allow early recognition of the deteriorating surgical patient? Critical Care Medicine. 2007;35: 402–409. doi: 10.1097/01.CCM.0000254826.10520.87 17205002 10.1097/01.CCM.0000254826.10520.87

[30] Weimar C, Mieck T, Buchthal J, Ehrenfeld CE, Schmid E, Diener H-C, et al Neurologic worsening during the acute phase of ischemic stroke. Arch Neurol. 2005;62: 393–397. doi: 10.1001/archneur.62.3.393 15767504 10.1001/archneur.62.3.393

[31] Diaz KM, Veerabhadrappa P, Kashem MA, Feairheller DL, Sturgeon KM, Williamson ST, et al Relationship of visit-to-visit and ambulatory blood pressure variability to vascular function in African Americans. Hypertens Res. 2012;35: 55–61. doi: 10.1038/hr.2011.135 21814215 10.1038/hr.2011.135 PMC3629695

[32] Hodgson JM, Woodman RJ, Croft KD, Ward NC, Bondonno CP, Puddey IB, et al Relationships of vascular function with measures of ambulatory blood pressure variation. Atherosclerosis. Elsevier Ltd; 2014;233: 48–54. doi: 10.1016/j.atherosclerosis.2013.12.026 24529122 10.1016/j.atherosclerosis.2013.12.026

[33] Tatasciore A, Zimarino M, Tommasi R, Renda G, Schillaci G, Parati G, et al Increased short-term blood pressure variability is associated with early left ventricular systolic dysfunction in newly diagnosed untreated hypertensive patients. Journal of Hypertension. 2013;31: 1653–1661. doi: 10.1097/HJH.0b013e328361e4a6 23811997 10.1097/HJH.0b013e328361e4a6

